# Supplementary material for: A Live Probiotic Vaccine Prototype Based on Conserved Influenza a Virus Antigens Protect Mice against Lethal Influenza Virus Infection
Source: Biomedicines. 2021 Oct 21;9(11):1515. doi: 10.3390/biomedicines9111515 (PMC8615285; doi:10.3390/biomedicines9111515)
Supplement: Supplementary file 1 [file biomedicines-09-01515-s001.zip › biomedicines-1420443-supplementary.pdf]

Supplementary material

d2-1

TGAGTGAACCACAGCCAGAAATTAATTCAAAAATGAGATCGATGAGAGCAGCTGGT

ATTGAGTTGAATGATACATTTCTATCTATTTACAGTTTAAATGGACAGTATCAGCA

ACGTGTGTCTTGGTATAATGACAATAATGAATCTGTCGGTGAACGTAATATTGATA

TGAGAGAATTTGTTGGGTATGAAAAAATGGGTAGCTTACCTTATTTTGTCACAACA

GATACAGCATGTGCAGAATACAAAGCTCCTGCGTTATCAACAAACAATTTAACTTC

AAAAGTAGTGGGAGGACGTGCAGAAAAGGCTTATAGCTCGAATGATCATTTCACCG

ATGTTGTAGGAGCTGATACTTATCACAGAAGTGGTGTAACGTATACGCTTCAAGGC

GCTTCCCCAACATTCATGATTGGCGCAAATACGAATAGTATGATGTTTAGCTTTGA

TACTGCATTGCTATGGACACCACAACCATCGAAGCCTACAAAAGAAGTGTTTAACA

AAGCTAATACTGAAGAGGCAGCACACAATATTGACAAAAAAGTGATTCCACAAGGA

TCAGATGTTTACTATCATATTCATCAAAAGTTTGATGCATTAACAGTCAACACAAT

GAACAAATACAAATCATTTAAAATCACTGATACCTTTGACAGCAAAAATTTTGATA

ha2

TGGTATCGGATGGGAAAAACTATGATGGCGCATTGCATATGCAATCTAGAGGCCTAT

TCGGGGCCATTGCCGGCTTCATTGAAGGGGGGTGGACAGGGATGGTAGATGGATGGT

ACGGTTATCACCATCAAAATGAGCAGGGGTCAGGATATGCAGCCGACCTGAAAAGCA

CACAAAATGCCATTGACAAGATTACTAACAAAGTAAATTCTGTTATTGAAAAGATGA

ATACACAGTTCACAGCAGTGGGTAAAGAGTTCAACCACCTGGAAAAGAGAATAGAGA

ATTTAAATAAAAAAGTTGATGATGGATTCCTGGACATTTGGACTTACAATGCCGAAC

TGTTGGTTCTATTGGAAAATGAAAGAACTTTGGACTACCACGATTCAAATGTGAAGA

ACTTGTATGAAAAGGTAAGAAACCAGTTAAAAAACAATGCCAAGGAAATTGGAAACG

GCTGCTTTGAATTTTACCACAAATGCGATAACACGTGCATGGAAAGTGTCAAAAATG

GGACTTATGACTACCCGAAATACTCAGAGGAAGCAAAATTAAACAGAGAAAAAATAG

A**TGGGGTAAAGCTGGAATCAACAGAATTC**

Figure S1. Sequence of the d2-1 region of the d2 gene encoding for N-(ha2) terminal part of D2 protein of *E.faecium* L3 strain. Primers that were used for amplification of the modified region of the enterococcal recombinant are shown in bold.

d2-1

TGAGTGAACCACAGCCAGAAATTAATTCAAAAATGAGATCGATGAGAGCAGCTGGT

ATTGAGTTGAATGATACATTTCTATCTATTTACAGTTTAAATGGACAGTATCAGCA

ACGTGTGTCTTGGTATAATGACAATAATGAATCTGTCGGTGAACGTAATATTGATA

TGAGAGAATTTGTTGGGTATGAAAAAATGGGTAGCTTACCTTATTTTGTCACAACA

GATACAGCATGTGCAGAATACAAAGCTCCTGCGTTATCAACAAACAATTTAACTTC

AAAAGTAGTGGGAGGACGTGCAGAAAAGGCTTATAGCTCGAATGATCATTTCACCG

ATGTTGTAGGAGCTGATACTTATCACAGAAGTGGTGTAACGTATACGCTTCAAGGC

GCTTCCCCAACATTCATGATTGGCGCAAATACGAATAGTATGATGTTTAGCTTTGA

TACTGCATTGCTATGGACACCACAACCATCGAAGCCTACAAAAGAAGTGTTTAACA

AAGCTAATACTGAAGAGGCAGCACACAATATTGACAAAAAAGTGATTCCACAAGGA

TCAGATGTTTACTATCATATTCATCAAAAGTTTGATGCATTAACAGTCAACACAAT

GAACAAATACAAATCATTTAAAATCACTGATACCTTTGACAGCAAAAATTTTGATA

lah+4m2e

TGGTATCGGATGGGAAAAACTATGATGGCGCATTGCATATGAATACACAGTTCACAG

CAGTAGGTAAAGAGTTCAACCACCTGGAAAAAAGAATAGAGAATTTAAATAAAAAAG

TTGATGATGGTTTCCTGGACATTTGGACTTACAATGCCGAACTGTTGGTTCTATTGG

AAAATGAAAGAACTTTGGACTACCACGATTCAAATGTGAAGAACTTATATGAAAAGG

TAAGAAGCCAGCTAAAAAACAATGCCGAATTCGTCGACAGCCTTCTAACCGAGGTCG

AAACACCTATCAGAAACGAATGGGGGTCCAGATCCAACGATTCAAGTGACGCTGCTG

CAGGTGGAGCAGCTAGCCTTCTAACCGAGGTCGAAACACCTATCAGAAACGAATGGG

GGTCCAGATCCAACGATTCAAGTGACGCTGCTGCACCAGGAGCAGCTAGTCTTCTAA

CCGAGGTCGAAACGCCTACCAGAAGCGAATGGGAGTCCAGATCCAGCGATTCAAGTG

ATGCTGCTGCAGGTGGAGCAGCTAGTCTTCTAACCGAGGTCGAAACGCCTACCAGAA

ACGAATGGGAGTCGCGCTC**GTCTGACAGTTCAGATGAATTC**

Figure S2. Sequence of the d2-1 region of the d2 gene encoding for N-(lah+4m2e) terminal part of D2 protein of *E.faecium* L3 strain. Primers that were used for amplification of the modified region of the enterococcal recombinant are shown in bold.
